# Recovery of neurological function of ischemic stroke by application of conditioned medium of bone marrow mesenchymal stem cells derived from normal and cerebral ischemia rats

**DOI:** 10.1186/1423-0127-21-5

**Published:** 2014-01-22

**Authors:** May-Jywan Tsai, Shen-Kou Tsai, Bo-Ruei Hu, Dann-Ying Liou, Shih-Ling Huang, Ming-Chao Huang, Wen-Cheng Huang, Henrich Cheng, Shiang-Suo Huang

**Affiliations:** 1 Neural Regeneration Laboratory, Center for Neural Regeneration, Department of Neurosurgery, Neurological Institute, Taipei Veterans General Hospital, No. 322, Section 2, Shih-Pai Road, Taipei City, Beitou District 112, Taiwan; 2 Vice Superintendent, Cheng Hsin General Hospital, Taipei, Taiwan; 3 Department and Institute of Pharmacology, School of Medicine, National Yang-Ming University, Taipei, Taiwan; 4 School of Medicine, National Yang-Ming University, Taipei, Taiwan; 5 Department of Pharmacology and Institute of Medicine, Chung Shan Medical University, Taichung, Taiwan; 6 Department of Pharmacy, Chung Shan Medical University Hospital, No.110, Sec. 1, Jianguo N. Road, Taichung City 402, Taiwan

**Keywords:** Mesenchymal stem cells, Conditioned medium, Neuronal cultures, Ischemic stroke, Neuroprotection, Cell surface markers

## Abstract

**Background:**

Several lines of evidence have demonstrated that bone marrow-derived mesenchymal stem cells (BM-MSC) release bioactive factors and provide neuroprotection for CNS injury. However, it remains elusive whether BM-MSC derived from healthy donors or stroke patients provides equal therapeutic potential. The present work aims to characterize BM-MSC prepared from normal healthy rats (NormBM-MSC) and cerebral ischemia rats (IschBM-MSC), and examine the effects of their conditioned medium (Cm) on ischemic stroke animal model.

**Results:**

Isolated NormBM-MSC or IschBM-MSC formed fibroblastic like morphology and expressed CD29, CD90 and CD44 but failed to express the hematopoietic marker CD34. The number of colony formation of BM-MSC was more abundant in IschBM-MSC than in NormBM-MSC. This is in contrast to the amount of Ficoll-fractionated mononuclear cells from normal donor and ischemic rats. The effect of cm of BM-MSC was further examined in cultures and in middle cerebral artery occlusion (MCAo) animal model. Both NormBM-MSC Cm and IschBM-MSC Cm effectively increased neuronal connection and survival in mixed neuron-glial cultures. *In vivo*, intravenous infusion of NormBM-MSC Cm and IschBM-MSC Cm after stroke onset remarkably improved functional recovery. Furthermore, NormBM-MSC Cm and IschBM-MSC Cm increased neurogenesis and attenuated microglia/ macrophage infiltration in MCAo rat brains.

**Conclusions:**

Our data suggest equal effectiveness of BM-MSC Cm derived from ischemic animals or from a normal population. Our results thus revealed the potential of BM-MSC Cm on treatment of ischemic stroke.

## Background

Ischemic stroke is one of the world’s fastest-growing diseases with high mortality and the leading cause of long-term disability worldwide [1]. There is no effective treatment available for either focal cerebral ischemia or global ischemic event apart from one recombinant tissue plasminogen activator (rt-PA) therapy directed at the dissolution of thrombi in affected blood vessel in adult following stroke [2]. A major limitation of r-tPA therapy for acute stroke is its narrow therapeutic window of 4.5 hours after stroke onset [1]. Beyond this timing of administration, rt-PA presents with deleterious side effects, in particular increase risk of intra-cerebral hemorrhage which can exacerbate stroke injury and counteract the benefits provided by reperfusion of the occluded artery in many patients [3].

There is increasing evidence that the transplanted bone marrow mesenchymal stem cell (BM-MSC) significantly promote functional recovery after central nervous system (CNS) damage in the animal models of various kinds of CNS disorders, including ischemic stroke [4]. In the ischemic stroke animal model, BM-MSC transplantation has been demonstrated to reduce cell apoptosis [5], induce angiogenesis [6], promote endogenous cell proliferation [7], and enhance axonal remodeling [8]. Recently, transplantation of BM-MSC was shown to achieve clinical efficacy in patients with ischemic stroke [9,10]. However, it is unclear what brings about the purported benefit from BM-MSC transplantation. The main goal of early BM-MSC studies in stroke was to differentiate into neurons and replace the injured neuron in infarct area [11,12]. However, very few transplanted cells were found in the brain and of these, only a small percentage cells expressed neuronal cell markers [13,14]. In addition, expression of neuronal cell markers did not indicate true differentiation and with neuronal cell function. Moreover, after BM-MSC transplantation, these cells, even differentiated cells, are very unlikely to have truly integrated into parenchymal tissue and form the complex connections that promote functional recovery [13]. Hence, it is unlikely that transplanted BM-MSC act to replace the damaged tissue. It is more feasible that BM-MSC might create a favorable environment for regeneration, and expression of beneficial bioactive factors. BM-MSC grafts have been shown to increase expression of several cytokines, neurotrophins and growth factors in ischemic brains. These include brain-derived neurotrophic factor (BDNF), glial cell-derived neurotrophic factor (GDNF), nerve growth factor (NGF), [13] , IGF-1 [15], stromal cell-derived factor-1 (SDF-1) [16], basic fibroblast growth factor (bFGF) and vascular endothelial growth factor (VEGF) [17], and all are responsible for the beneficial effects of BM-MSC against ischemic stroke on brain protection and tissue regeneration.

Recent studies suggested that hypoxic preconditioning of BM-MSC significantly enhanced homing of transplanted cells to the ischemic region and effectively promoted the regenerative capability and therapeutic potential of BM-MSC for the treatment of ischemic stroke [18,19]. However, it remains elucidated whether BM-MSC derived from healthy donors or stroke patients provides equal therapeutic potential. The present work aims to characterize BM-MSC obtained from normal healthy rats and cerebral ischemia rats, and examine the effects of their conditioned medium (Cm) on ischemic stroke animal model.

## Methods

### Reagents and antibodies

Cultured medium, fetal bovine serum (FBS), serum-free supplements and antibiotics were purchased from Gibco (Carlsbad, CA, USA). Antibodies used in this study are listed as follows: rabbit anti-betaIII tubulin (Upstate Biotechnology, Lake Placid, NY, USA), mouse anti-ED1 (CD68) (Serotec, England, UK), goat anti-doublecortin (DCX, Chemicon, Merck Millipore), mouse anti-BrdU (Chemicon, Merck Millipore), mouse anti-CD90-PE (BD biosciences), mouse anti-CD44-FITC (BD biosciences), mouse anti-CD34-FITC (BD biosciences); mouse anti-CD29-FITC (BD biosciences). Unless stated otherwise, all other chemicals were purchased from Sigma-Aldrich Co.

### Animal surgery and treatment

Adult male Long Evan (LE) rats (6–8 weeks old; 250–350 g) were obtained from National Laboratory Animal Center, Taiwan. All efforts were taken to minimize animal suffering during and following surgery. Middle cerebral arterial occlusion (MCAo) surgery was used for creating focal cerebral ischemic injury. Focal cerebral ischemic injury was produced in the right lateral cerebral cortex by permanent ligation of MCA with 10–0 monofilament nylon. Both common carotid arteries were clamped for 60 minutes and then reperfusion of flow was confirmed visually during surgery before closure of the wound. BM-MSC Cm was intravenously infused to MCAo rats immediately after blood reperfusion. The functional motor deficits in experimental rats were quantified at 1, 3, 7 days post-injury. Measures of brain infarction and histochemical staining were conducted at 1 week after right MCA occlusion.

### Isolation and expansion of mesenchymal stem cells from bone marrow

Bone marrows were aspirated from femur bones of normal or post 1 week-MCAo adult LE rats. Bone marrow cells were flushed out from femurs with phosphate buffered saline (PBS; GIBCO) and filtered through nylon cloths (70 μm sieve). The filtered cells were collected by centrifugation (326 × g for 10 minutes), resuspended and diluted with equal volume of Dulbecco’s modified Eagle’s medium containing F12 (DMEM/F12). The resulted cell suspension was layered onto Ficoll-paque solution (1.077 g/mL) and centrifuged to deplete the residues of red blood cells, platelets, and plasma. Ficoll-fractionated mononuclear cells were recovered from the gradient interface. The isolated cells were washed once, seeded in 75 cm^2^ flask (Falcon) and maintained in DMEM/F12 supplemented with 10% fetal calf serum (FCS), 100 U/mL penicillin and 100 μg/mL streptomycin at 37°C in a water-saturated atmosphere of 5% CO_2_/95% air. Non-adherent cells were removed at 2 days after initial seeding. Cultures developed colonies of fibroblast-like cells (CFU-f) within 2 weeks. The attached cells at about 80% confluence were subcultured and expanded. Cultured cells were phenotypically characterized by flow cytometric analysis. The proliferative activities of cultured cells were investigated by pulsing subconfluent cells with 10 uM 5-bromo-2’-deoxyuridine (BrdU; Sigma) for 3 hours. The cells were then fixed and immunostained with anti-BrdU, whereas nuclei were counterstained with Hoechst 33342 (Sigma).

### Immunophenotypic analyses of expressed antigens on cell surface

For further characterization, cell surface antigen phenotyping was performed on isolated and expanded bone marrow cells were detected at passages 0 to 3 by flow cytometric analysis. The adherent cells were harvested by treatment of 5 mM EDTA in PBS solution. Cells were stained for 1 hour on ice with fluorescein isothiocyanate (FITC)- or phycoerythrin (PE)-conjugated anti-marker monoclonal antibodies. Antibodies used for specific surface markers included hematopoietic lineage early marker (CD34), Thy-1 (CD90), integrin (CD29) and CD44. The stained cells were subsequently analyzed by fluorescence-activated cell sorter (FACS Calibur flow cytometer; BD bioscience) using a 525 nm band-pass filter for green FITC fluorescence and a 575 nm band-pass filter for red PE fluorescence.

### Preparation of conditioned medium of bone marrow mesenchymal stem cells (BM-MSC Cm)

Second to third passages BM-MSC were processed for BM-MSC Cm collection. When cultures reached ~80% confluence, cultures were washed trice with PBS and refilled with DMEM/F12 supplemented with 2% LE rat serum. The cm was collected after incubation with BM-MSC for 24 hours. BM-MSC Cm were further concentrated with a centrifugal filter device (5 kDa cut-off, Amicon Ultra, Millipore). The resulted cm (~10 fold concentrated) of BM-MSC were preserved at −80°C until use.

### Behavioral test

Functional behaviors in rats were tested at 1, 3, 5 and 7 post-injury or before sacrifice. Contralateral motor deficits in the rat forelimbs due to the damage of stroke–affected brain were evaluated using grasping power test [20,21]. The grasping power test is a modification of the method of Bertelli and Mira [20] using a commercial grip-strength meter (Grip-strength- meter 303500, TSE systems Corp) for rats. Both forepaws were tested, testing one forepaw at a time. The untested forepaw was temporarily prevented from grasping by wrapping it with adhesive tape, and the tested forepaw was kept free. The rats were allowed to grasp the bar while being lifted by the tail with increasing firmness until they loosened their grip, and the grasping power was scored.

### Morphological analysis

Rats were sacrificed at one week after ischemia-reperfusion for infarct volume analysis (by 2,3,5-triphenyltetrazolium chloride (TTC) staining) and immune histochemistry (IHC). For TTC staining, rat brains were quickly removed, placed to a brain matrix slicer (Jacobowitz Systems, Zivic-Miller Laboratories Inc., Allison Park, PA, USA) and sectioned into 2 mm coronal slices. The resulted slices were stained with 2% TTC for 30 min and fixed in 10% buffered formalin solution overnight. TTC positive staining, indicating viable tissues, was used to verify successful stroke and treatment. Infarct volumes (negative TTC staining area) were analyzed using AIS imaging research software (Imaging Research Inc., St. Catharines, Ontario, Canada). The area of infarction was measured by subtracting the area of the non-lesioned ipsilateral hemisphere from that of the contralateral side plus negative TTC staining area. Infarct volume was calculated as the sum of infarct area per slice multiplied by slice thickness [22]. Both the surgeon and image analyzer operator were blinded to the treatment given each animal. The conditioned medium is blind to the surgeon and operator too. For fluorescence immunocytochemical staining, the tissues were post-fixed with 4% paraformaldhyde, processed in series with 15% and 30% sucrose and finally embedded in OCT compound (Sakura Fine Technical, Tokyo, Japan). Tissues were cut into serial 10 μm sections with a cryostat. Immunocytochemical staining was performed on serial sections as described in our previously published papers [23]. Images of immunoreactive cells in brain sections were obtained with a fluorescent microscope equipped with fluorescence optics and with a CCD camera.

### Cortical neuronal cultures

Cortical neuronal cultures were prepared from the cerebrocortical regions of Sprague–Dawley (SD) rat fetuses at gestation 15–17 days as described in Tsai et al. [24-26]. In brief, fetal cortexes were dissociated with mixtures of papain/protease/deoxyribonuclease I (0.1%: 0.1%: 0.03%). Cultures were plated onto poly-D-lysine coated multi-well plates and maintained in Dulbecco’s modified Eagle’s medium (DMEM, Gibco) with 10% FBS at 37°C in a water-saturated atmosphere of 5% CO_2_ and 95% mixed air. Second day after cell seeding, aliquots of NormBM-MSC Cm or IschBM-MSC Cm was added to neuronal culture and incubated for 3 days. Culture medium was then collected for lactate dehydrogenase (LDH) assay and cells were fixed for immunofluorescent staining. For quantitative analysis of neurite density, 20 images per condition were obtained. Neurite density was analyzed using ImageJ software (NIH systems). A commercial kit (CellTiter 96 Aqueous; Promega Corporation) was used for determining the extent of cell survival as cytosolic LDH release in cultured medium. Activity of LDH in the medium was determined by the reduction of MTS tetrazolium into colored formazan products and measure absorbance at 490 nm.

### Statistical analysis

All measurements were performed blind to each group. Experimental data were expressed as the mean of independent values ± s.e.m. and were analyzed using one-way analysis of variance (ANOVA) followed by Bonferroni’s *t*-test. *P* values less than 0.05 were considered statistically significant.

## Results

### Characterization of BM-MSC cultured from normal or ischemic rats

**Figure 1:**
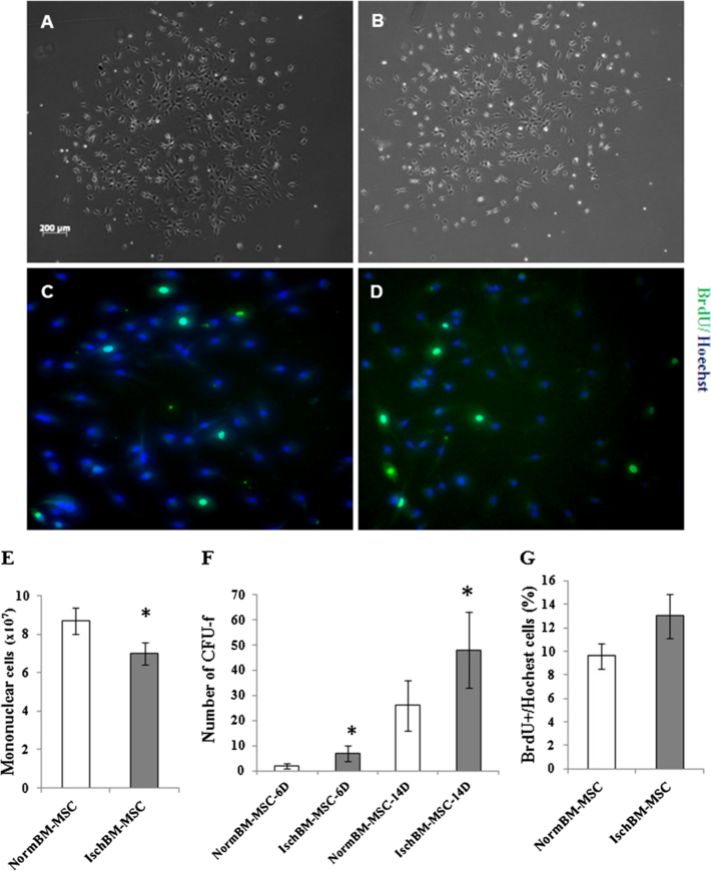
**Characterization of BM-MSC cultured from normal or ischemic rats. (A,B)** round-shape colonies of fibroblastic like NormBM-MSC and IschBM-MSC, respectively. **(C,D)** Proliferative activities of NormBM-MSC and IschBM-MSC were stained with anti-BrdU labeled FITC (green) and Hoechst (blue) incorporation. Magnification: 200× **(E)** Ficoll-fractionated mononuclear cell numbers from bone marrows of normal or ischemic rats. n = 13 ~ 16; *P < 0.05 **(F)** Numbers of colony forming unit-fibroblast (CFU-f) from bone marrow cultures at 6 and 14 days *in vitro*. n = 4 per group; *p < 0.05 IschBM-MSC vs. NormBM-MSC **(G)** Quantitative cell proliferation of NormBM-MSC and IschBM-MSC.

We first characterized the cells and cultures prepared from normal or post-MCAo LE rat bone marrow. This data was calculated from Ficoll-separated bone marrow cells from 13–16 rats. After Ficoll-paque centrifugation, mononuclear cells of bone marrow were aspirated from the density interface (1.077 g/ml, lymphocytic layer), pelleted and washed twice with PBS. The resulted cells were counted before seeding for adherent cultures (BM-MSC). Figure 1E shows that Ficoll-fractionated mononuclear cells from normal rats were significantly more abundant than that from ischemic rats (p < 0.05). BM-MSC, also referred to as colony-forming fibroblast (CFU), are able to form round-shape colonies of fibroblastic like cells, namely CFU-f [27,28]. Figure 1A and B shows typical colonies of NormBM-MSC and IschBM-MSC with similar morphology. Colonies appear as a central core of round cells surrounded by more elongated cells at the periphery. Because the number of colonies is an index of MSC functional capacity, clones of >50 fibroblasts (as fibroblastic colony) from NormBM-MSC and IschBM-MSC were scored at 6 days and 14 days after initial seeding. As the quantitative results, these expanded BM-MSC increased the number of colonies over time (Figure 1F). Interestingly, IschBM-MSC possessed higher frequency of CFU-f than NormBM-MSC (day 6: 1.75 ± 1.70 and 7.50 ± 3.10 colonies per 75 T flask in NormBM-MSC and IschBM-MSC, respectively; day 14: 25.75 ± 10.60 and 48.25 ± 14.40 colonies per T75 flask in NormBM-MSC and IschBM-MSC, respectively; n = 4 per group; p < 0.05). To investigate whether proliferative ability of BM-MSC rendering different CFU-f in NormBM-MSC and IschBM-MSC, *in vitro* BrdU incorporation was conducted in cells after subculture. As the immunocytochemical result, the number of BrdU-positive cells increased in the IschBM-MSC group (Figure 1D), with a definite trend, compared with the NormBM-MSC group (Figure 1C). Primary cultures of BMSC from normal or ischemic rats reach ~80% confluence at definite periods, in vitro BrdU incorporation was analyzed after cell passage. The quantitative ratio of BrdU (+) cells/Hoechst in both cultures were shown in Figure 1G. These results indicate that IschBM-MSC can be expanded rapidly *ex vivo* and might be more available to provide cell therapy for stroke.

### Characterization surface protein expression of BM-MSC cultured from normal or ischemic rats

**Figure 2:**
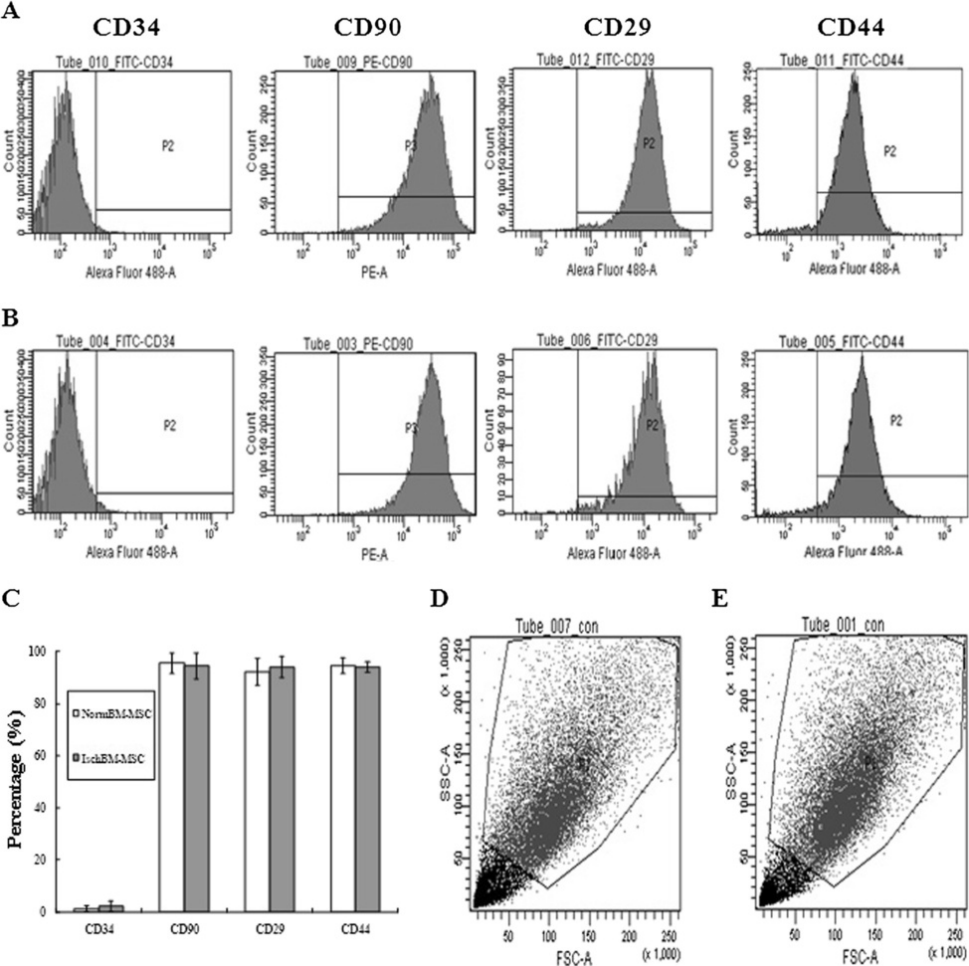
**Flow cytometry analysis of bone marrow mesenchymal stem cells (BM-MSC) from normal and cerebral ischemic rats. (A)** analysis of cell surface markers in NormBM-MSC **(B)** analysis of cell surface markers in ischBM-BMSC **(C)** comparative analysis of cell surface markers in NormBM-MSC and IschBM-MSC **(D-E)** FACS analyses of total cell populations in cultured NormBM-MSC **(D)** and IschBM-MSC **(E)**. Cells (passages 1-3) were lifted, labeled with antibodies against the indicated antigens, and analyzed by flow cytometry. Only representative examples featuring antigen expression profiles are shown. Abbreviations: FITC, fluorescein isothiocyanate; PE, phycoerythrin.

For further characterization of the BM-MSC, surface protein expression of BM-MSC of isolated and expanded cells was carried out using fluorescence-activated cell sorting (FACS) analysis at cell passages 0 to 3. Figures 2A and B showed that more than 98% of isolated NormBM-MSC or IschBM-MSC expressed typical MSC marker protein, β1-integrin (CD29) and Thy1 (CD90). Approximate >90% CD44-positive cells existed in NormBM-MSC and IschBM-MSC population. By contrast, both cultures failed to express immunoreactivity (IR) to CD34, a surface marker for early hematopoietic stem cells. Figure 2C shows that there was no significant difference in cytometric analysis of cell surface markers between NormBM-MSC and IschBM-MSC at the second passage. Figure 2D and E show similar total cell populations by FACS analyses in cultured NormBM-MSC (D) and IschBM-MSC (E). We also evaluated the soluble factors released from BM-MSC by zymography and western blot analysis. Additional file 1: Figure S1 (C) shows western blot identification of 5 factors, including aFGF, TIMP1, IGFBP4, VEGF (in non-reduced form) and SCF, which exist at similar levels between NormBM-MSC Cm and IschBM-MSC Cm. Furthermore, SDS gel and zymographic results (detecting activities of MMP2 and MMP9) in both Cm have similar patterns.

### Effects of Cm from NormBM-MSC and IschBM-MSC on neuronal survival

**Figure 3:**
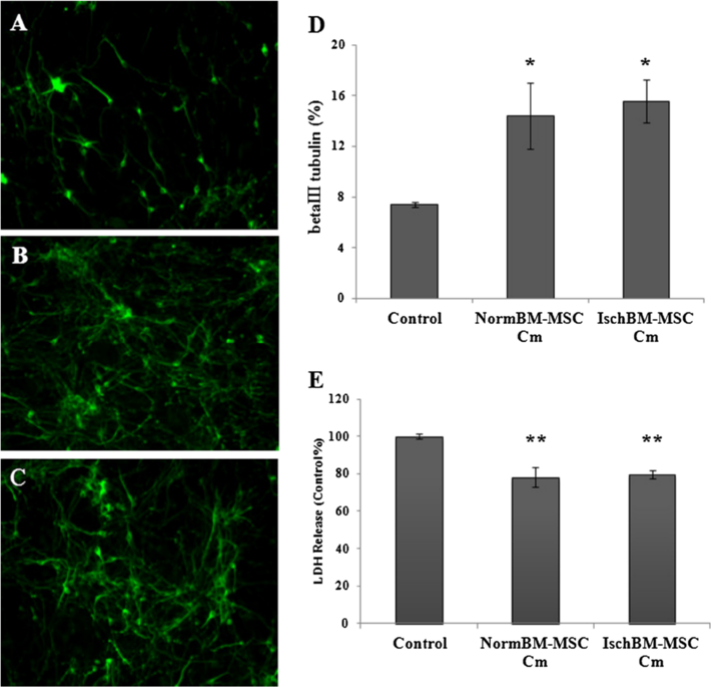
**Conditioned medium from NormBM-MSC or IschBM-MSC increased cortical neuronal survival by enhancing neuronal connection and decreasing LDH release. (A)** Neuronal cells, non-treated control. **(B)** NormBM-MSC Cm-treated neuronal cells **(C)** IschBM-MSC Cm-treated neuronal cells **(D)** βIII tubulin positive signal, as percentage of total area, quantified by Image J, in cortical neuronal cells. **(E)** LDH release to medium in cortical neuronal cells. Three days after treatment, cortical cell cultures were immunostained with anti-βIII-tubulin, a neuronal marker and medium were collected for LDH assay. Data are presented as means ± SEM from 8–10 independent experiments done in duplicate. *, **P < 0.05, 0.01 Treatment versus control. Magnification 200× **(A-C)**.

Three days after treatment, cortical cell cultures were immunostained with anti-βIII-tubulin, a neuronal marker and medium were collected for LDH assay. Compared to control cultures, both NormBM-MSC Cm and IschBM-MSC Cm increased neuronal connection, as shown in Figure 3A-C. Quantitative results of betaIII tubulin immunoreactivity by Image J further shows that neuronal density was significantly enhanced by the treatment of NormBM-MSC Cm or IschBM-MSC Cm (Figure 3D). Release of cytosolic lactate dehydrogenase (LDH) is a hallmark of destroyed cells. Figure 3E shows that the percentage of cytosolic LDH release was significantly reduced in the treatment with NormBM-MSC Cm and IschBM-MSC Cm compared with medium control (100%). Thus, these results suggest that NormBM-MSC Cm and IschBM-MSC Cm promoted cell integrity and might decrease cell susceptibility after CNS injury.

### Effects of Cm from NormBM-MSC and IschBM-MSC on rat ischemic stroke

**Figure 4:**
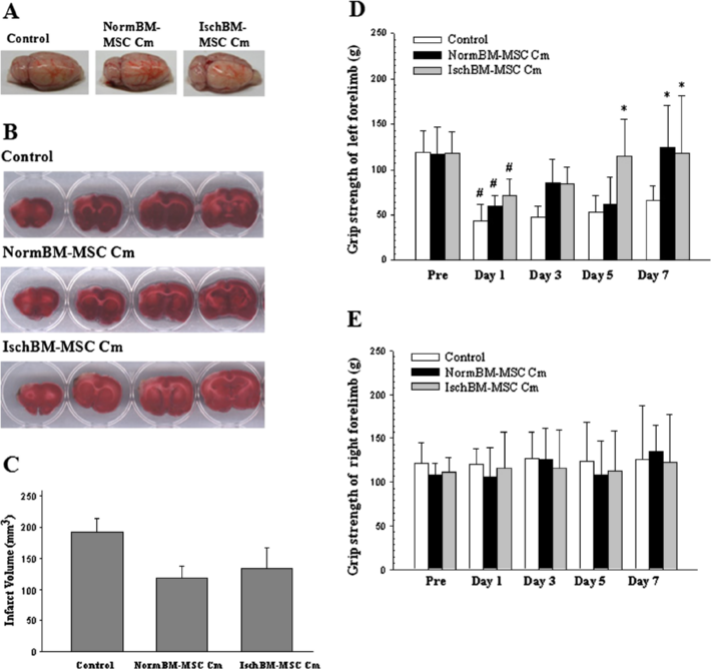
**Effects of administration of conditioned medium from NormBM-MSC and IschBM-MSC on infarct volume and functional behavior in MCAo rats. (A)** The right lateral view of MCAo rat brains showing the stroke outcome from different treatments, **(B)** The infarct volumes of MCAo rat brains evaluated at 7 days postinjury using TTC staining analysis **(C)** Quantitative results of TTC staining of MCAo rat brains from different treatment, These results were taken as the mean ± S.D. from 8 ~ 15 rats/group (Control n = 8, NormBM-MSC Cm n = 15, IschBM-MSC n = 9), **(D)** Grip strength of left forelimbs (g) assessed using grasping power test on stroke-affected side (#p < 0.05 versus pre-MCAo, *p < 0.05 versus Control), **(E)** Grip strength of right forelimbs (unaffected side). Data were given as the mean ± S.D. from 6 ~ 7 rats/group (7 rats for control, 6 rats for NormBM-MSC Cm and 6 rats for IschBM-MSC Cm).

We determined the effect of cm from NormBM-MSC and IschBM-MSC in reducing brain infarction after ischemic stroke in rats. Results showed that NormBM-MSC Cm and IschBM-MSC Cm decreased brain infarction caused by 60-min MCAo and following reperfusion for 7 days based on quantification of TTC staining of brain slices; however, statistical significance was not achieved (Figure 4A-C). In addition to the extent of brain infarction, behavioral deficits in the MCAo-treated rats with or without cm were examined. The grasping power of the left forepaws of rats before MCAo or that of the right forepaws of rats before or at the end of the 7 days post-MCAo (Figure 4D-E) did not differ among these groups. Interestingly, MCAo significantly decreased grasping power that was improved by administration of NormBM-MSC Cm and IschBM-MSC Cm at the end of the 7 days post-MCAo (Figure 4D).

### Effects of Cm from NormBM-MSC and IschBM-MSC on neurogenesis *in vivo*

**Figure 5:**
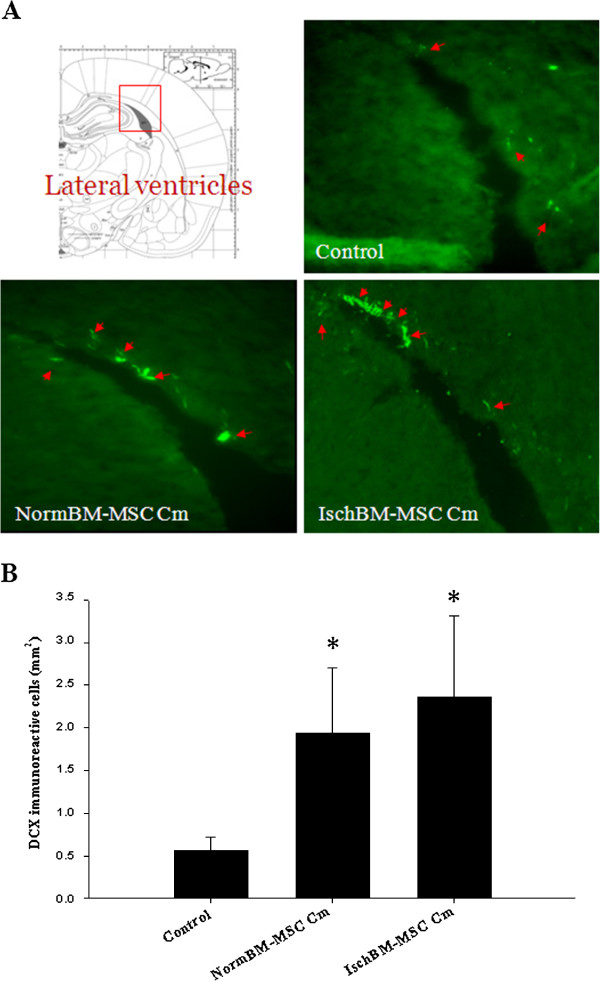
**Promoted sprouting of neuronal progenitor cells in the lateral ventricles of MCAo rats treated with NormBM-MSC Cm and IschBM-MSC Cm. (A)** The schematic picture displays the assessed lateral ventricle near hippocampus; Coronal sections of brain tissues from different treatment were immunostained with anti-doublecortin (DCX; green). DCX-positive cells were located along with lateral ventricles in the group given with BM-MSC Cm; magnification 200× (**B)**: The immunoreactive area of DCX-positive cells was determined using Image-Pro Plus software. Representative data were taken as the means ± S.D. of three repetitions.

Results that NormBM-MSC Cm and IschBM-MSC Cm improved functional outcome without a significant reduction in infarct volume suggest mechanisms other than neuroprotection may contribute to the observed functional improvement. To examine the effect of NormBM-MSC and IschBM-MSC on the survival or proliferation of progenitor cells and/or immature neurons in the ischemic brain, coronal sections of brain tissue were immuostained for doublecortin (DCX) at seven days after MCAo. DCX is a marker for neuronal progenitor cells and immature neurons to evaluate neuronal plasticity. Figure 5A and 5B showed that application of NormBM-MSC Cm and IschBM-MSC Cm enhanced the extent of DCX-positive cells in the lateral ventricular area of stroke-affected hemispheres, indicating a heightened generation of neural progenitor cells.

### Effects of Cm from NormBM-MSC and IschBM-MSC on microglia/macrophage infiltration *in vivo*

**Figure 6:**
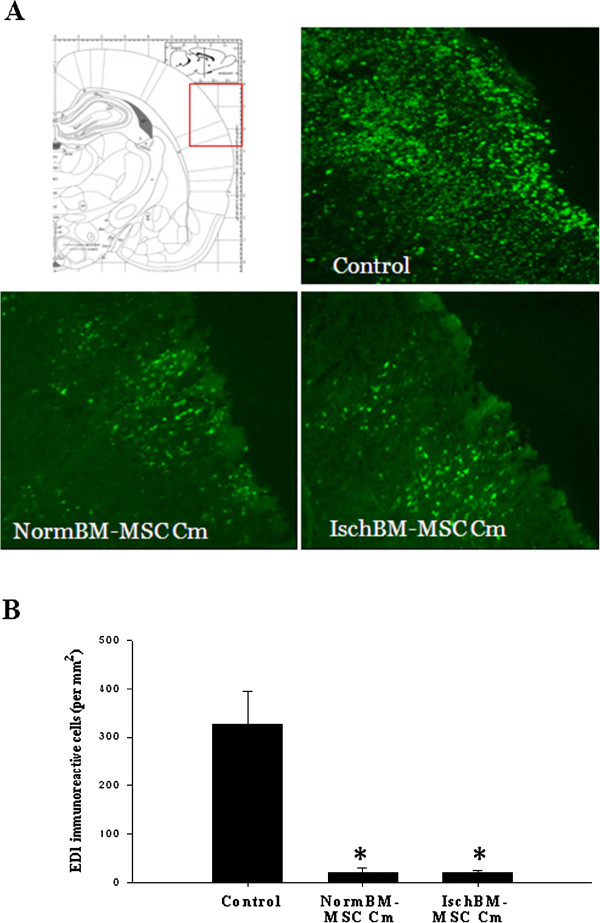
**Attenuated infiltration of microglia/macrophage in the cortical regions of MCAo rats given NormBM-MSC Cm and IschBM-MSC Cm. (A)** The illustration indicated the cortical region of the brain tissue was examined. Coronal sections of brain tissue were immunostained with anti-ED1 antibody labeled FITC. Immunofluorescent showed that ED-1 positive cells were abundant in the ischemic cortex on day 7 after MCAo (Control), whereas ED-1 positive cells were fewer in the group given NormBM-MSC Cm or IschBM-MSC Cm. ED-1 positive cells were absent in the unaffected hemisphere after MCAo. **(B)** The numbers of ED1 immunoreactive cells were determined using Image-Pro Plus software. Representative data were taken as the means ± S.D. of three repetitions.

We performed fluorescence immunohistochemistry for ED1, a marker for activated microglia and macrophage, in coronal section of brain tissues at seven days after MCAo. Immunostaining results showed that a large number of ED1-positive cells were located on the ischemic core and the ischemic boundary zone of the stroke-affected cortices at seven days after MCAo in the medium control group (Figure 6A). By contrast, only a few ED1-positive cells were observed in the ischemic cortex in the group receiving NormBM-MSC Cm or IschBM-MSC Cm (Figure 6A). Quantitative analysis exactly confirmed that MCAo rats receiving NormBM-MSC Cm or IschBM-MSC Cm significantly reduced accumulation of ED1-positive cells (Figure 6B). Thus, these observations suggest that the intervention with NormBM-MSC Cm and IschBM-MSC Cm minimizes pro-inflammatory cascades during cerebral ischemia, at least in part, via attenuating microglia/macrophage infiltration.

## Discussion and conclusions

In current study, we are the first to compare the effects of treatment of ischemic stroke with NormBM-MSC Cm or IschBM-MSC Cm. We presented experimental evidence supporting the notion that administration of NormBM-MSC Cm or IschBM-MSC Cm may reduce the extents of brain injury *in vivo* and *in vitro*. Furthermore, our data suggests that enhancement of neurogenesis and attenuation of microglia/macrophage infiltration may contribute to the underlying beneficial effect of NormBM-MSC Cm and IschBM-MSC Cm.

We first compared the characteristics of NormBM-MSCs and IschBM-MSCs. In the present study, we found that the typical colonies of NormBM-MSC and IschBM-MSC with similar morphology and there was no significant difference in the expression of cell surface markers between NormBM-MSC and IschBM-MSC. Ficoll-fractionated mononuclear cells from normal rats were significantly more abundant than that from ischemic rats. However, IschBM-MSC possessed higher frequency of CFU-f than NormBM-MSC. These different characteristics did not influence the effect of NormBM-MSC Cm and IschBM-MSC Cm on cortical neuron. *In vitro* experiment demonstrates that both NormBM-MSC Cm and IschBM-MSC Cm significantly increased cortical neuronal survival and promoted neuronal connection compared with medium control. These results suggest that both NormBM-MSC Cm and IschBM-MSC Cm promoted cell integrity and might decrease cell susceptibility after CNS injury. There is no significant difference between NormBM-MSC Cm and IschBM-MSC Cm.

We further examined the effects of NormBM-MSC Cm and IschBM-MSC Cm on rat subjected to ischemic stroke. *In vivo* data shows that both NormBM-MSC Cm and IschBM-MSC Cm improved neurological outcome but did not reduce the ischemic lesion. Recently, Zacharek *et al.,* elucidated that treatment of stroke with BM-MSCs derived from stroke rats were better than normal population due to the enhanced increasing of angiogenesis and arteriogenesis via Ang1/Tie2 system as well as neurological outcomes [29]. However, we found that the recovery of neurological function after ischemic stroke was not significant different between NormBM-MSC Cm and IschBM-MSC Cm. Accumulating evidence has suggested that BM-MSC promote endogenous neurogenesis to improve functional recovery after stroke in rats [7,30]. Our data shows that NormBM-MSC Cm and IschBM-MSC Cm substantially increased neuronal progenitor cells (DCX-positive cells) surrounding lateral ventricle in stroke-affected hemisphere. Intriguingly, NormBM-MSC Cm and IschBM-MSC Cm also significantly attenuated microglia/macrophage infiltration in the ischemic brain.

Together, results shown in the current study indicate that treatment with NormBM-MSC Cm and IschBM-MSC Cm after stroke significantly improved functional outcome but did not substantially decrease cerebral infarction. Enhancement of neurogenesis and attenuating microglia/macrophage infiltration may contribute to the observed improvement of functional outcome. Our findings indicate the potential of BM-MSC Cm on treatment of ischemic stroke. Patient’s age and morbidity may influence the BM-MSC effects [31,32]. In addition, the autologous BM-MSC requires harvesting bone marrow cells from patients with stroke and subsequent culturing for several days [33]. Allogeneic cells can be obtained from young, healthy donors, *ex vivo* expanded and stored for immediate use when needed [32,33]. Furthermore, our data suggests that the efficiency is equal by using BM-MSC Cm derived from patients with stroke or from a normal population. Our results point out that the BM-MSC Cm from an ischemic animal is not better than from normal one for the treatment of stroke. The present article adds to the issues under consideration that BM-MSC behaves as extracorporeal bioreactors to produce bioactive factors in the form of several effective compounds contained in conditioned medium that may become a novel therapeutic strategy for clinical use on ischemic stroke. Our results conclude that use of BM-MSC Cm from an ischemic animal for the treatment of stroke has equal efficiency as compare with BM-MSC Cm from a normal one.

## Competing interests

The authors declare that they have no competing interests.

## Authors’ contributions

MJT, BRH, SLH and DYL carried out cell culture and animal studies. MJT, SKT, MCH and WCH participated in the design of the study and drafted the manuscript. HC and SSH conceived of the study, participated in its design and coordination, and collectively prepared the manuscript. All authors have read the final manuscript.

## Supplementary Material

Additional file 1: Figure S1 Analysis and Identification of soluble factors released to conditioned media of NormBM-MSC and IschBM-MSC. (a) Representative gel electrophoretic analysis of Cm from NormBM-MSC and IschBM-MSC (15 ul Cm/lane) (b) Representative gelatin-Zymographic analysis of Cm from NormBM-MSC and IschBM-MSC (5 ul Cm/lane) (c) Representative western blot analysis of Cm from NormBM-MSC and IschBM-MSC (7.5 ul Cm/lane). SCF stands for stem cell factor; TIMP 1 stands for tissue inhibitor of metalloproteinase 1. VEGF is the abbreviation of vascular endothelial growth factor; IGFBP4 is the abbreviation of insulin-like growth factor-binding protein 4. aFGF stands for acidic fibroblast growth factor. Click here for file
