# Supplementary figures and images for: Recovery of neurological function of ischemic stroke by application of conditioned medium of bone marrow mesenchymal stem cells derived from normal and cerebral ischemia rats
Source: J Biomed Sci. 2014 Jan 22;21(1):5. doi: 10.1186/1423-0127-21-5 (PMC3922747; doi:10.1186/1423-0127-21-5)

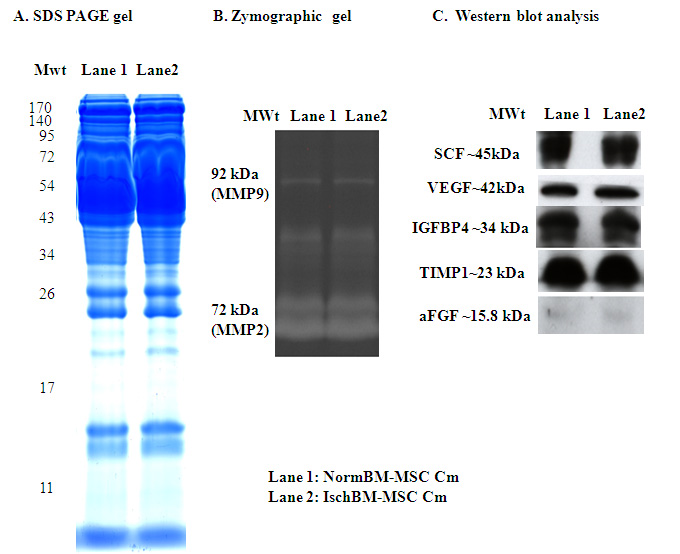

Supplement: Additional file 1: Figure S1 — Analysis and Identification of soluble factors released to conditioned media of NormBM-MSC and IschBM-MSC. (a) Representative gel electrophoretic analysis of Cm from NormBM-MSC and IschBM-MSC (15 ul Cm/lane) (b) Representative gelatin-Zymographic analysis of Cm from NormBM-MSC and IschBM-MSC (5 ul Cm/lane) (c) Representative western blot analysis of Cm from NormBM-MSC and IschBM-MSC (7.5 ul Cm/lane). SCF stands for stem cell factor; TIMP 1 stands for tissue inhibitor of metalloproteinase 1. VEGF is the abbreviation of vascular endothelial growth factor; IGFBP4 is the abbreviation of insulin-like growth factor-binding protein 4. aFGF stands for acidic fibroblast growth factor. [file 1423-0127-21-5-S1.jpeg]
